# Effects of Health-Promoting Lifestyles in Midlife on Cognitive Functioning in Later Life

**DOI:** 10.1093/geroni/igab046.1197

**Published:** 2021-12-17

**Authors:** Eunsaem Kim, Yunhwan Lee, Jonggak Shin, Gyeonghui Kim, Jihye Yoon

**Affiliations:** 1 Ajou University School of Medicine, Suwon, Kyonggi-do, Republic of Korea; 2 Korea Employment Information Service, EUMSEONG-GUN, Ch'ungch'ong-bukto, Republic of Korea

## Abstract

Maintaining cognitive function in later life is key to healthy aging because cognitive impairments compromise everyday functional abilities, impeding independent living. Numerous studies have discovered early life experiences and lifestyle behaviors over the lifespan to have substantial influences on cognitive functioning with age. Especially, subtle brain changes related to dementia occur as early as midlife, and lifestyle factors in midlife influence neuropathological development, suggesting that midlife is a critical period for preserving cognitive health in later life. This study investigated the association between lifestyle behaviors in midlife and cognitive performance in later life using 12-year follow-up data from the Korean Longitudinal Study on Aging (KLoSA). Cognitive function was assessed with the Harmonized Cognitive Assessment Protocol (HCAP) for KLoSA. Eight thousand respondents from the KLoSA sample were administered HCAP neuropsychological tests. Hierarchical multiple regression analyses were used to examine whether health-promoting lifestyles at baseline (2006) predicted cognitive function in 2018 after controlling for health-related covariates. We identified a positive influence of health-protective behaviors (non-smoking, moderate drinking, regular exercise, weight management, and health screening) at baseline on language abilities in 2018 (β = .05, p < .05). In addition, health-promoting behaviors covering interpersonal relationships, social engagement, optimistic outlook, and positive attitudes at baseline were predictive of language abilities (β = .08, p < .01), executive function (β = .06, p < .01), and the visuospatial ability (β = .06, p < .05) in 2018. This study highlights the importance of midlife health-promoting lifestyles in maintaining cognitive health in later life.

